# Overproduced bone marrow neutrophils in collagen‐induced arthritis are primed for NETosis: An ignored pathological cell involving inflammatory arthritis

**DOI:** 10.1111/cpr.12824

**Published:** 2020-06-22

**Authors:** Danyi Xu, Yiming Lin, Jinming Shen, Jie Zhang, Jinghua Wang, Yuwei Zhang, Hong Zhang, Longgui Ning, Peihao Liu, Sha Li, Hang Zeng, Jin Lin, Chaohui Yu

**Affiliations:** ^1^ Department of Rheumatology The First Affiliated Hospital College of Medicine Zhejiang University Hangzhou China; ^2^ Department of Gastroenterology The First Affiliated Hospital College of Medicine Zhejiang University Hangzhou China; ^3^ Department of Orthopaedics The First Affiliated Hospital Zhejiang University of Traditional Chinese Medicine Hangzhou China

**Keywords:** bone marrow lesion, G‐CSF, neutrophil extracellular traps, rheumatoid arthritis

## Abstract

**Objectives:**

Bone marrow edema is a universal manifestation of rheumatoid arthritis (RA), and its pathological essence is a bone marrow lesion (BML) formed by various bone marrow (BM) immune cells. Neutrophils play an important role in inflammatory arthritis, but the role and mechanism of neutrophils in BML are not clear.

**Materials and methods:**

Granulocyte colony‐stimulating factor (G‐CSF) −/− mice and wild type (WT) C57BL/6 mice were immunized for collagen‐induced arthritis (CIA). Histological scores of arthritis were evaluated. Immunohistochemistry staining with anti‐Ly6G was conducted. Neutrophil extracellular traps (NETs) in joint sections were determined by immunofluorescence staining. BM neutrophils were isolated for flow cytometry and NETosis induction in vitro.

**Results:**

Histological study showed significant neutrophil infiltrations in BML of CIA mice. Inhibition of BM neutrophil production by G‐CSF knock out can obstruct the induction of BML and CIA. In addition to abundant infiltrated NETs intra‐articular, remarkable NETosis primed BM neutrophils were infiltrated in BML of CIA mice, which was positively related to bone erosion. Neutrophils derived from G‐CSF−/− mice have diminished ability of NETs formation in vitro, while G‐CSF induction can enhance its capacity of NETs formation.

**Conclusions:**

We propose for the first time that the overproduced BM neutrophils in CIA mice are primed for NETosis in a G‐CSF dependent manner, and these pathogenic cells may have an important role in inflammatory arthritis. Blocking this pathological process could be a potential strategy for the treatment of RA.

## 1 INTRODUCTION

Rheumatoid arthritis (RA) is a complex autoimmune disease involving immune cells, synovial cells, osteoclasts, and other cells. T, B lymphocytes, dendritic cells, macrophages, and other immune cells play an important role in the occurrence and development of RA, but the exact pathogenesis of RA is still unclear.^1^ The majority of researchers have focused on lesions in the synovium, cartilage, and bone cortex, but in fact, immune cells in the subchondral bone marrow (BM) also play a role in RA.^2^

Bone marrow edema (BME) is a non‐specific imaging manifestation of the high signal lesions in the BM region on the fat‐suppressed image of MRI.^3^ BME is common in inflammatory joints of RA and can appear in the early stage of disease.^4^ Studies have shown that BME score is closely related to cartilage and bone erosion, and is a sensitive indicator that reflects the risk and progress of cartilage and bone erosion in RA.^5^ Besides, BME was correlated with anti‐citrullinated protein antibodies (ACPA) levels in RA patients.^6^ Therefore, controlling BME may have potential benefits for the treatment and prognosis of RA.

From a pathological point of view, the essence of BME is that normal adipose tissue is replaced by inflammatory cells mainly composed of T, B lymphocytes.^7^, ^8^ This pathological manifestation is called bone marrow lesions (BMLs).^9^ McQueen et al proposed the pathogenesis hypothesis of BML in RA, suggesting that pathological cells migrate from subchondral bone to synovium to play a role.^10^ However, there are few studies in this field, and the exact role and mechanism of BML in RA is still unclear.

Neutrophils are differentiated from precursor cells in the BM and released into the peripheral blood. In the inflammatory state, BM neutrophil production increases, which can migrate and infiltrate into the joint cavity of RA patients under chemotaxis, and further participate in arthritis by phagocytosis, degranulation, and production of reactive oxygen species (ROS).^11^ In recent years, the discovery of neutrophil extracellular traps (NETs) has further elucidated the role of neutrophils in the pathogenesis of RA.^12^ Whether neutrophils are involved in BML of RA has not been reported yet. The purpose of this study was to determine the role of neutrophils in BML of inflammatory arthritis and its possible pathways and mechanisms by a murine model of RA.

## 2 MATERIALS AND METHODS

### 2.1 Mice

Granulocyte colony‐stimulating factor(G‐CSF) −/− mice (a kindly gift from Professor Jiong Chen, Laboratory of Biochemistry and Molecular Biology, School of Marine Sciences, Ningbo University, Ningbo, China) were derived on the 129Sv × B6 background and backcrossed onto B6 mice for more than 20 generations. G‐CSF−/− and wild type (WT) C57BL/6 mice were maintained at 23 ± 2°C in a 12 h light/12 h dark cycle at the Experimental Animal Center in Zhejiang Province (Hangzhou, China). All mice used in this study were males aged 10‐14 weeks. All animal studies were performed in adherence to the guidelines approved by the Animal Care and Use Committee of the First Affiliated Hospital, College of Medicine, Zhejiang University.

### 2.2 Collagen‐induced arthritis

Collagen‐induced arthritis (CIA) which is the major murine model of RA was induced as described previously.^13^ Briefly, mice were immunized intradermally in the proximal tail with 100 μg of chicken type II collagen (2 mg/mL, 20012; Chondrex, Inc) emulsified in 250 μg of Freund's complete adjuvant (5 mg/mL, 7023; Chondrex, Inc). A booster injection was given 21days after the first immunization. The incidence and severity of arthritis were assessed from the second immunization up to the termination of the experiment. A clinical arthritis score from 0 to 4 was given for each limb with a maximal score of 16 for each mouse (0: no erythema and swelling; 1: slight edema and erythema limited to the tarsals or ankle; 2: slight edema and erythema spreading from the ankle to the tarsal bone; 3: moderate edema and erythema from the ankle to the metatarsal joints; 4: severe edema and erythema from the ankle to the entire paw).^14^ The mice were sacrificed at day 51 and the ankle joints were collected.

### 2.3 Histological examination

The ankle joins were fixed in 4% paraformaldehyde overnight, decalcified in ethylenediaminetetraacetic acid (EDTA) for 4 weeks, processed and embedded in paraffin. Joint sections were stained with hematoxylin and eosin. Histological score of arthritis was evaluated by two investigators separately who were blinded to grouping information, according to a previously described scoring system on the extent of synovitis, pannus formation, bone and/or cartilageerosion, as following criteria: 0: no signs of inflammation; 1: mild inflammation with hyperplasia of the synovial lining layer, minimal without cartilageerosion; 2 to 4: increasing degrees of inflammatory cell infiltrate, or cartilage and bone erosion.^15^

### 2.4 Immunohistochemistry

For immunohistochemistry, paraffin‐embedded slices on slides were deparaffinized with xylene and rehydrated. Antigens were unmasked by boiling in EDTA antigen retrieval buffer pH 9.0. Endogenous peroxidases were blocked with 3% hydrogen peroxide solution and then slides were blocked with 10% normal goat serum. Sections were then incubated with anti‐Ly6G (Rat, 1:300, Ly6G 1A8, BE0075‐1; Bioxcell). Labeling was detected with an HRP labeled goat anti‐Rat secondary antibody and DAB. Slides were counterstained with Harris hematoxylin. The immunohistochemical staining was observed and photographed with a light microscope. The number of positively stained cells was measured using the Image‐Pro Plus 6.0 image analysis software.

### 2.5 Immunofluorescence staining for joint sections

After sectioning to 4 µm, the joint section was blocked with 10% normal goat serum for 45 minutes. The sections were incubated with an anti‐citrullinated Histone H3 antibody (Rabbit, 1:250, citrulline R2 + R8 + R17, ab5103; Abcam) and anti‐Ly6G antibody (1:300) or anti‐myeloperoxidase (MPO) (mouse, 2D4, 1:50, ab90810; Abcam). Isotype control antibodies were used. After three washes, Alexa 488‐goat anti‐rabbit (A‐11034; Invitrogen) to recognize the anti‐Histone H3 and Cy3‐goat anti‐rat (A10522; Invitrogen) to recognize the Ly6G were both incubated for 1 hour at room temperature. A 4',6‐diamidino‐2‐phenylindole (DAPI) nuclear stain was applied for 10 minutes followed by three washes of phosphate buffer saline (PBS) to remove residual dye. Slides were analyzed and imaged with a confocal microscope (Nikon Eclipse Ti). NETs or neutrophil initiated NETosis were identified according to the colocalization of DAPI, Ly6G, and NETs marker cit‐H3.

### 2.6 Isolation of mouse BM neutrophils

Bone marrow cells were flushed from the tibias and femurs of WT and G‐CSF−/− mice into Roswell Park Memorial Institute (RPMI) media‐1640. Neutrophils were purified from BM as described previously.^16^ 10^7^‐10^8^ neutrophils were obtained from each mouse and the cell survival rate was more than 95% counted by trypan blue stain. The purity of isolated neutrophils was more than 90% assessed by Wright‐Giemsa stain.

### 2.7 Flow Cytometry

Single‐cell suspensions of BM cells were re‐suspended in RPMI‐1640 for staining. Cell surface staining was performed to evaluate frequencies of mature BM neutrophils using antibodies against CD45, CD11b and Ly6G directly labeled with APC, PE‐Cy7 or FITC (BD pharmingen). Appropriate isotypes were used as antibody controls. Stained cells were analyzed using a FACSCalibur flow cytometer (BD Biosciences).

### 2.8 NETosis induction in vitro

Neutrophils obtained from WT and G‐CSF−/− mice were seeded at a density of 10^5^ cells/well in a 24‐well cell culture plate with polylysine‐coated coverslips for 30min. Then, the cells were exposed to PBS ormice serum or phorbol 12‐myristate 13‐acetate (25 nmol/L, PMA; Sigma)with or without G‐CSF (100 ng/mL, Recombinant Human G‐CSF; Proteintech) for 3 hours at 37°C with 5% CO_2_. For G‐CSF induction, BM neutrophils were primed with G‐CSF at 100 ng/mL for 1 hour before PMA stimulation.

### 2.9 Immunofluorescence staining for BM neutrophils

Cells were fixed in 4% paraformaldehyde for 15 minutes and permeated by 0.2% Triton X‐100/PBS for 10 minutes. Cells were incubated with 10% normal goat serum for 40 minutes and then incubated with NETs markers anti‐citrullinated Histone H3 (Rabbit, 1:250, citrulline R2 + R8 + R17, ab5103; Abcam) and anti‐MPO (mouse, 2D4, 1:50, ab90810; Abcam) or with anti‐Neutrophil Elastase (Rabbit, 1:100, ab21595; Abcam) in a humidified chamber overnight at 4°C. Isotype control antibodies were used. After washing with PBS three times, cells were incubated with secondary antibodies conjugated with fluorescein for 1 hour. The cells were then incubated with DAPI for 5 minutes to stain the DNA. The cells were analyzed and imaged with a confocal microscope (Nikon Eclipse Ti). Neutrophils release NETs were counted manually and shown as the number of neutrophils release NETs per field for at least 6 fields from different regions. NETs were identified as the cloud‐like or filamentous structures with colocalization of DAPI and NETs markers.

### 2.10 Statistical analysis

All statistical analyses were performed using GraphPad Prism (version 7.0). Between‐ group difference was analyzed by Student's *t* test or Mann–Whitney *U* test. The correlation between groups was analyzed using the Pearson correlation coefficient. A value of *P* < .05 was considered statistically significant.

## 3 RESULTS

### 3.1 Significant neutrophil infiltration in BML of CIA mice

**FIGURE 1:**
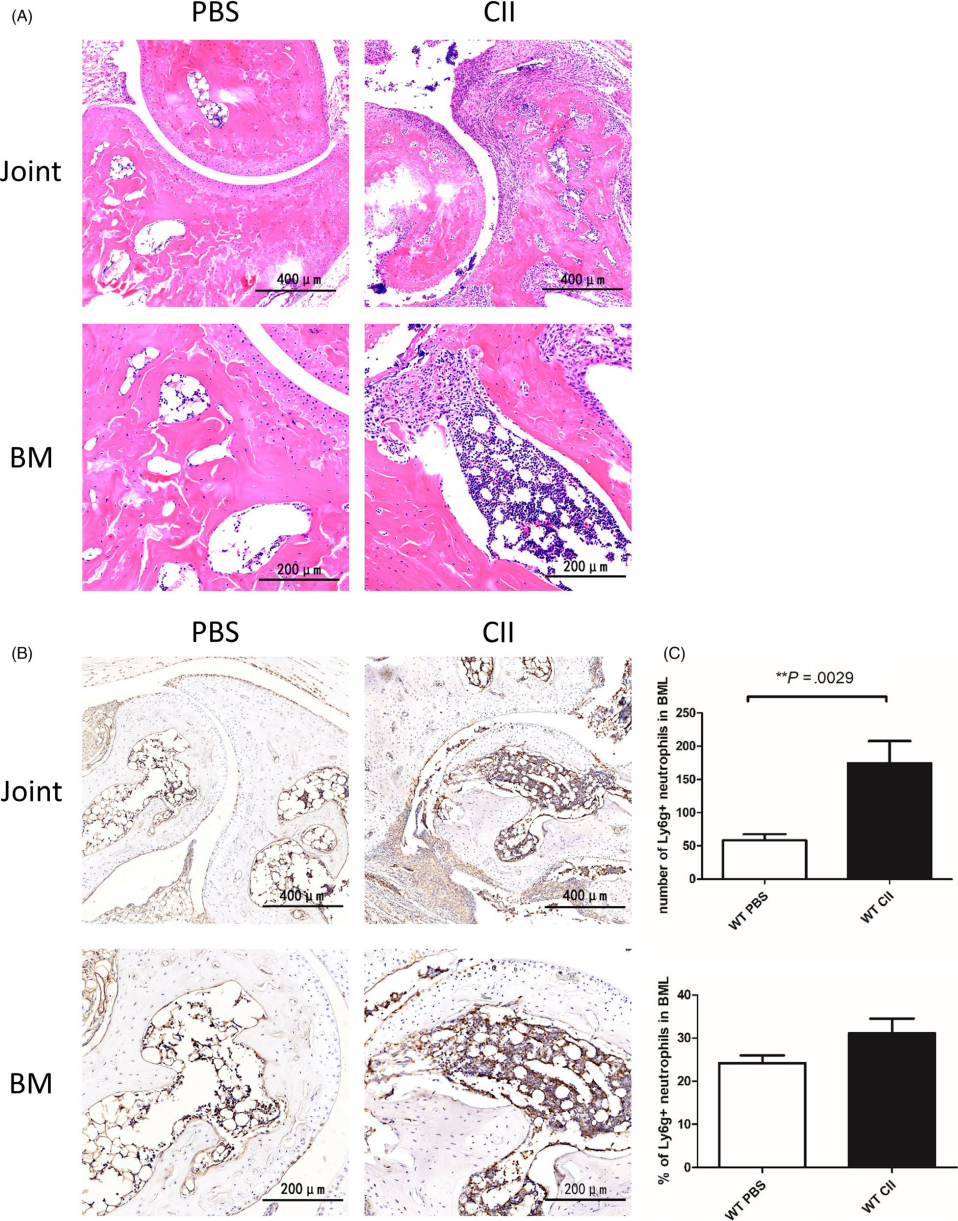
Collagen‐induced arthritis (CIA) mice showed significant neutrophil infiltration in bone marrow lesion (BML). A, Representative images of hematoxylin and eosin‐stained sections of arthritis and subchondral BML obtained from the ankle joints of CIA and normal control mice. B, Representative pictures of neutrophils infiltrating the ankle joints and subchondral bone marrow (BM) of CIA and normal control mice, by Ly6G staining (in brown). C, The number and percentage of subchondral BM Ly6G‐ positive cells per field in CIA vs normal control mice were analyzed by Image‐pro plus 6.0, as determined by two‐tailed, unpaired Student's *t* test (mean ± SEM). Results shown are representative for five fields in different regions of each section and two independent experiments with 8 individual mice per group

Previous studies have reported that the main histological change of RA subchondral BML is the infiltration of a large number of lymphocytes,^7^, ^8^ but whether there is infiltration of neutrophils has not been reported in the literature. Therefore, we induced CIA model and determined it through pathological methods. HE staining showed that CIA mice had marked BML in which normal adipose tissues were replaced by infiltrated immune cells, while the BM of the normal control mice were mainly adipose tissue (Figure 1A). Immunohistochemical staining using the murine neutrophil surface marker Ly6G showed that Ly6G positive neutrophils infiltrated into the articular cavity, synovium and articular cartilage of CIA mice and also in subchondral BML significantly, in contrast, no neutrophils were found in the joints and BM of control mice (Figure 1B,C). These results show that there were significant neutrophil infiltrations in BMLs of CIA mice. We speculate that the intensive neutrophil infiltration in BML may be due to the excessive production of BM neutrophils under the stimulation of inflammation.

### 3.2 Inhibition of BM neutrophil production by G‐CSF knock out obstruct the induction of CIA

**FIGURE 2:**
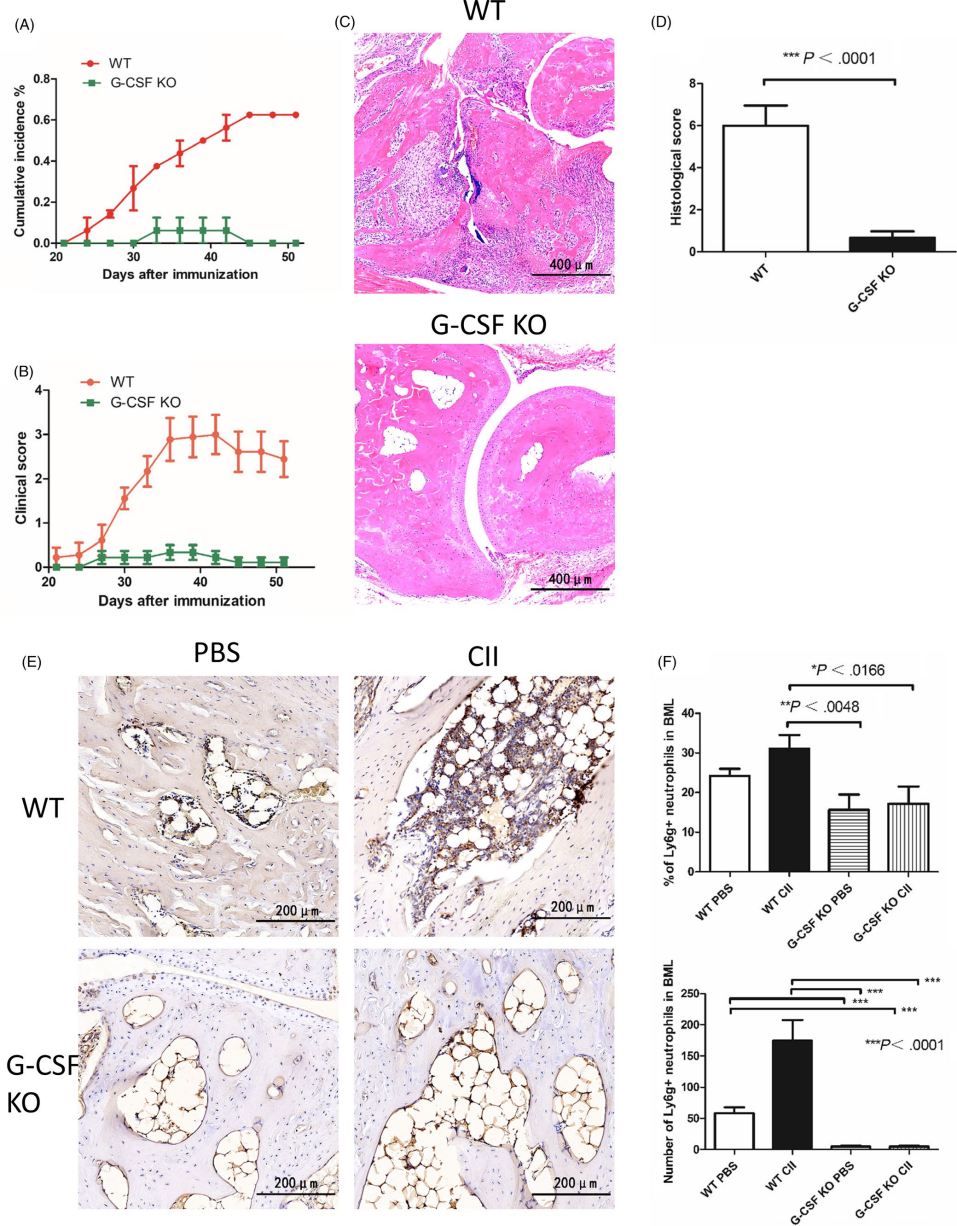
Suppression of bone marrow (BM) neutrophil production by granulocyte colony‐stimulating factor (G‐CSF) knockout can significantly obstruct the induction of collagen‐induced arthritis (CIA). A, The cumulative incidence of arthritis and (B) clinical scores (mean ± SEM) in immunized WT and G‐CSF−/− mice during days 21‐51 of CIA induction. Results shown are from 8 individual mice per group and two independent experiments. C, Representative histopathological sections of ankle joints from immunized WT and G‐CSF−/− mice by hematoxylin and eosin staining. D, The semi‐quantitative histological score was evaluated and compared, as determined by two‐tailed, unequal variance Student's *t* test (mean ± SEM). Results shown are from 8 individual mice per group and two independent experiments. E, Representative immunohistochemical staining of Ly6G in subchondral BM. Brown staining indicates Ly6G positive cells. F, The percentage and number of Ly6G positive cells per field in subchondral BM from normal control or immunized WT and G‐CSF−/− mice were enumerated for at least five different fields of each section by Image‐pro plus 6.0, as determined by two‐tailed, unpaired Student's *t* test (mean ± SEM). Results shown are from 8 individual mice per group and two independent experiments

The differentiation and maturation of BM neutrophils are mainly regulated by G‐CSF. G‐CSF is not only a hematopoietic factorbut also a proinflammatory factor by promoting BM granulopoiesis under inflammatory stimulation.^17^ CIA model of WT and G‐CSF−/− mice were used to further confirm the effect of excessive BM neutrophil production in BML information and its role in the development of inflammatory arthritis. The cumulative incidence of arthritis and the mean arthritis score are dramatically reduced in G‐CSF−/− mice compared with WT mice (Figure 2A‐C). On the histologic evaluation of the joints, there was extensive infiltration of inflammatory cells, synovitis and severe cartilage and bone erosionsin WT mice, while the joints from G‐CSF−/− mice were normal (Figure 2D,E). In the subchondral BM, WT mice showed significant BMLs with heavy neutrophil infiltrations, while G‐CSF−/− mice had no BML formation and neutrophil infiltration. There were even more BM neutrophils in WT normal control mice than in immunized G‐CSF−/− mice (Figure 2F). Therefore, excessive production of BM neutrophils is necessary for BML formation and it plays an important role in the pathogenesis of CIA, this process is G‐CSF dependent.

### 3.3 BM neutrophils involved in inflammatory arthritis through NETosis

**FIGURE 3:**
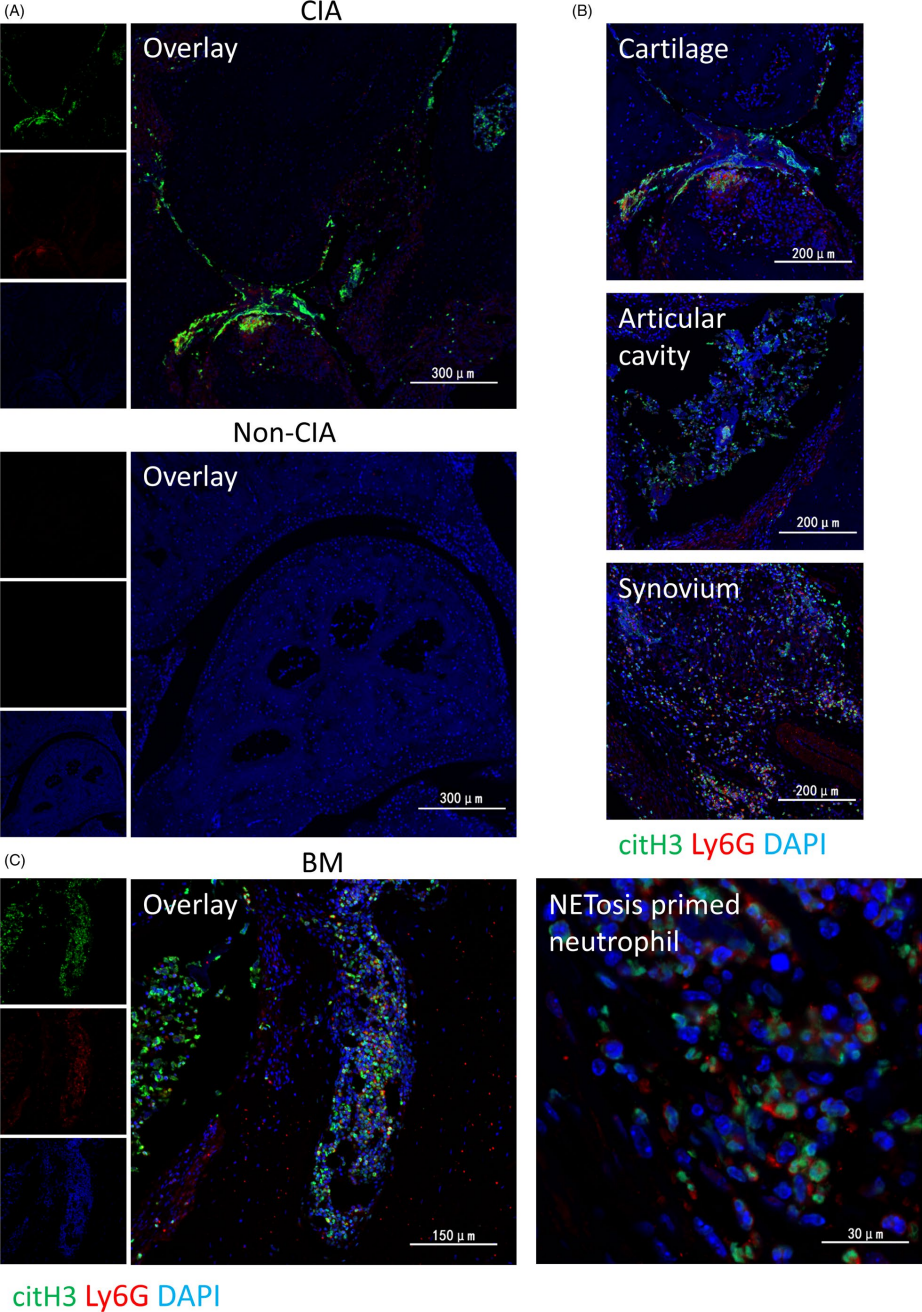
Significant NETosis primed phenomenon of bone marrow (BM) neutrophils, in addition to neutrophil extracellular traps (NETs) intra‐articular. A, Representative confocal images of NETs formation in joints of collagen‐induced arthritis (CIA) or non‐CIA by immunofluorescent staining. B, Representative confocal images of NETs formations in cartilage, articular cavity, synovium. C, Representative confocal images of neutrophils primed for NETosis in subchondral BM of WT CIA mice and magnified NETosis primed neutrophils

**FIGURE 4:**
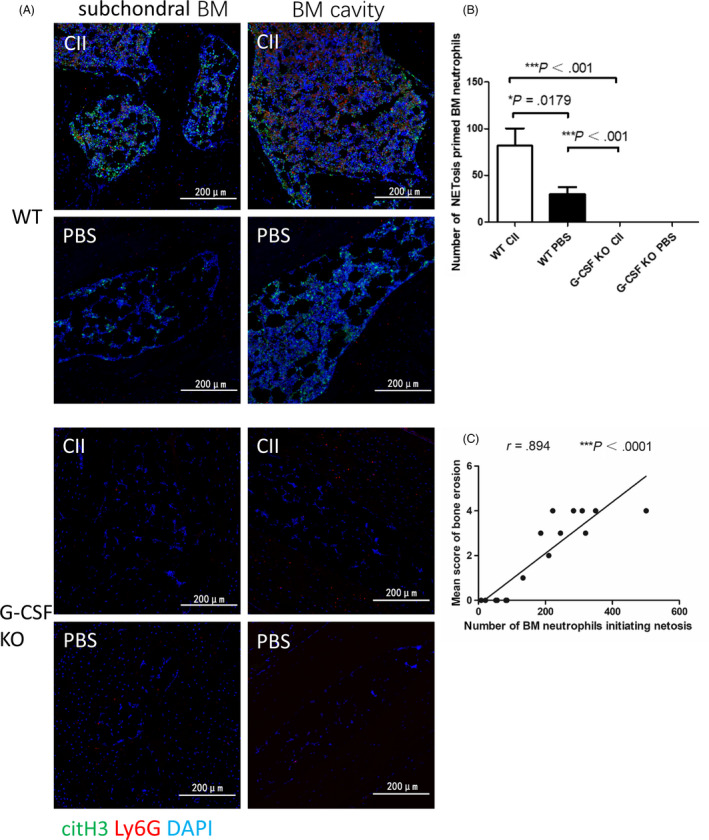
Bone marrow (BM) neutrophils may be involved in inflammatory arthritis through NETosis. A, Representative confocal images of neutrophils primed for NETosis in subchondral BM and BM cavity of immunized or normal control WT and granulocyte colony‐stimulating factor (G‐CSF)−/− mice. B, The number of BM neutrophils primed for NETosis per field in immunized or normal control WT compare to G‐CSF−/− mice, as determined by Mann‐Whitney *U* test (mean ± SEM). C, Correlation between the number of NETosis primed BM neutrophils and the pathological score of bone erosion in WT mice. Results shown are representative of three different fields of subchondral BM and three different fields of BM cavity in each section from 8 individual mice per group and 2 independent experiments were repeated. BM Neutrophils primed for NETosis were defined as colocalized cit‐H3, Ly6G, and DNA. All pictures were taken by confocal microscopy (Nikon Eclipse Ti)

Previous studies have shown that BME is associated with ACPA antibody,^6^ meanwhile,the citrullinated histones generated by neutrophils in the process of NETs formation are the real antigens of ACPA.^18^ Therefore, we assume that BM neutrophils are involved in the onset of arthritis through NETosis. The role of NETs in a variety of diseases, including RA, has been confirmed, but current studies are limited to NETosis in peripheral blood or affected organs, and it is unclear whether BM neutrophils are also involved in NETosis. Therefore, we further investigated the NETosis phenomenon in the joints and subchondral BM of CIA mice. In order to analyze the formation of NETs in CIA, we used anti‐citrullinated histone H3 antibody, a NETs‐specific marker, to stain the joint tissues by immunofluorescence. There was a large amount of NETs infiltration in the joints of CIA mice, distributed mainly in the articular cavity, articular cartilage, synovial tissue, etc, which was consistent with the location of neutrophil infiltration (Figure 3A,B). Just as we expected, a large number of anti‐citrullinated histone H3‐positive neutrophils wereinfiltrated in BML of CIA mice, but no definite NETs structures observed. (Figure 3C). However, there was no intra‐articular NETosis phenomenon of WT normal control mice, immunized or normal control G‐CSF−/− mice. (Figure 3A). In contrast, there were limited anti‐citrullinated histone H3‐positive BM neutrophils in WT normal control mice while absolutely no anti‐citrullinated histone H3‐positive BM neutrophils in G‐CSF−/− mice (Figure 4A,B). Furthermore, there was a significant positive correlation between the number of anti‐citrullinated histone H3‐positive BM neutrophils and the pathological score of bone erosion in WT mice (Figure 4C). These results suggest that these overproduced neutrophils in the BM of CIA have undergone citrullination of histones. The different expression of citrullinated histone in BM between WT normal control mice and G‐CSF−/− mice suggests that G‐CSF deficiency may blocking the citrullination of histones in BMneutrophils. So BM neutrophils may involve in inflammatory arthritis through NETosis, and G‐CSF deficiency may inhibit inflammatory arthritis via blocking NETosis.

### 3.4 BM neutrophils are primed for NETosis

**FIGURE 5:**
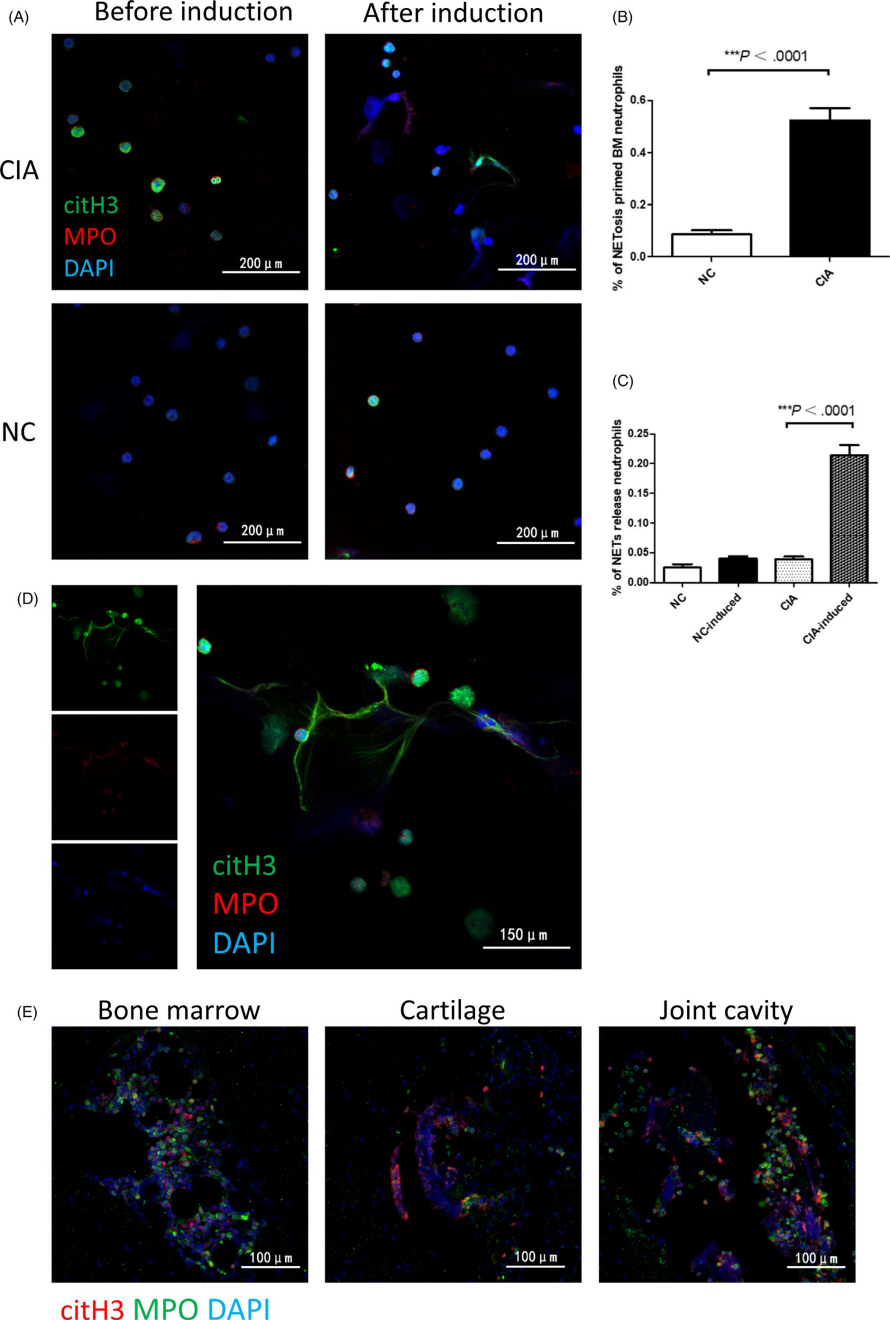
Bone marrow (BM) neutrophils are primed for NETosis. A, Representative confocal images of BM neutrophils isolated from collagen‐induced arthritis (CIA) mice and normal control mice before and after induction of serum. B, Percentage of BM neutrophils primed for NETosis per field in neutrophils from CIA mice as compared to neutrophils from normal control mice before induction, (C) Percentage of BM neutrophils forming neutrophil extracellular traps (NETs) per field from normal control and CIA mice before induction as compared to that of after induction with their corresponding serum, as determined by two‐tailed, unequal variance Student's *t* test (mean ± SEM). Results shown are representative of at least eight fields in different regions for each condition, and representative of two independent experiments. D, Magnified representative confocal images of BM neutrophils from CIA mice forming NETs in vitro after induction. E, Magnified representative confocal images of NETosis primed neutrophils in BM and NETs formation in cartilage and joint cavity

Histone citrullination is a key step in NETosis, but it does not represent the formation of NETs. To further clarify whether BM neutrophils have initiated NETosis or only primed for NETosis, we performed immunofluorescence staining on joint tissue (citrullinated histone H3, MPO, DNA), meanwhile, we isolated BM neutrophils from CIA mice and control mice and used their autologous serum to induce NETs formation in vitro. The results showed that citrullinated histone H3 was positive in BM neutrophils in CIA mice, but MPO was not present in the nucleus, and morphologically, there was no significant NETs formation before induction with the autologous mice serum (Figure 5A). BM neutrophils were primed for NETosis from CIA mice, but not in normal control mice (Figure 5B). Significant NETs formations were observed in BM neutrophils of CIA mice induced with their serum in vitro (Figure 5A,C,D), while in control mice, there was no significant NETosis (Figure 5A,C). In contrast, there were citrullinated histone H3, MPO, and DNA co‐localized NETs in the joints of CIA mice, mainly distributing in the joint cavity, articular cartilage, etc (Figure 5E). These results indicate that BM neutrophils are primed for NETosis. Once released into the periphery and subjected to chemotaxis of inflammation, these NETosis primed cells can infiltrate into inflammatory joints and form NETs under inflammatory stimulation, promoting the development of arthritis.

### 3.5 G‐CSF affects the ability of BM neutrophils to form NETs in vitro

**FIGURE 6:**
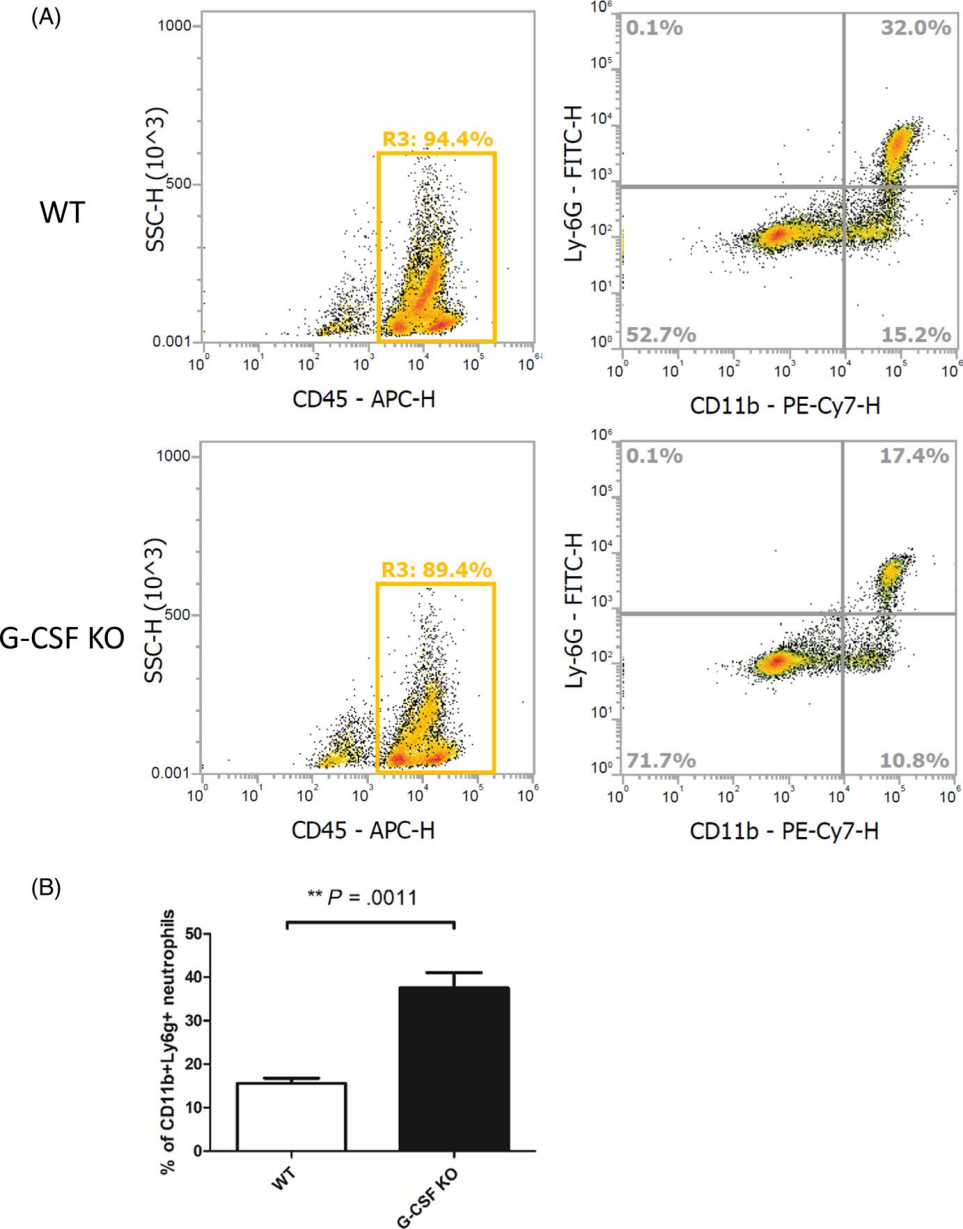
Impaired maturity of bone marrow (BM) neutrophils from granulocyte colony‐stimulating factor (G‐CSF)−/− mice. A, Representative dot plots of the relative proportion of mature neutrophils. B, The percentage of mature neutrophils relative to the total number of WT BM cells as compared to G‐CSF−/− BM cells, as determined by two‐tailed, unequal variance Student's *t* test (mean ± SEM). Results shown are from 8 individual mice per group and two independent experiments

To further investigate the effect of G‐CSF on the formation of NETs in BM neutrophils, we extracted and isolated BM neutrophils from WT and G‐CSF−/− mice to induce NETs in vitro. The lower proportion of CD11b + Ly6G + mature neutrophils in G‐CSF−/− BM was confirmed by flow cytometry (Figure 6A,B).

**FIGURE 7:**
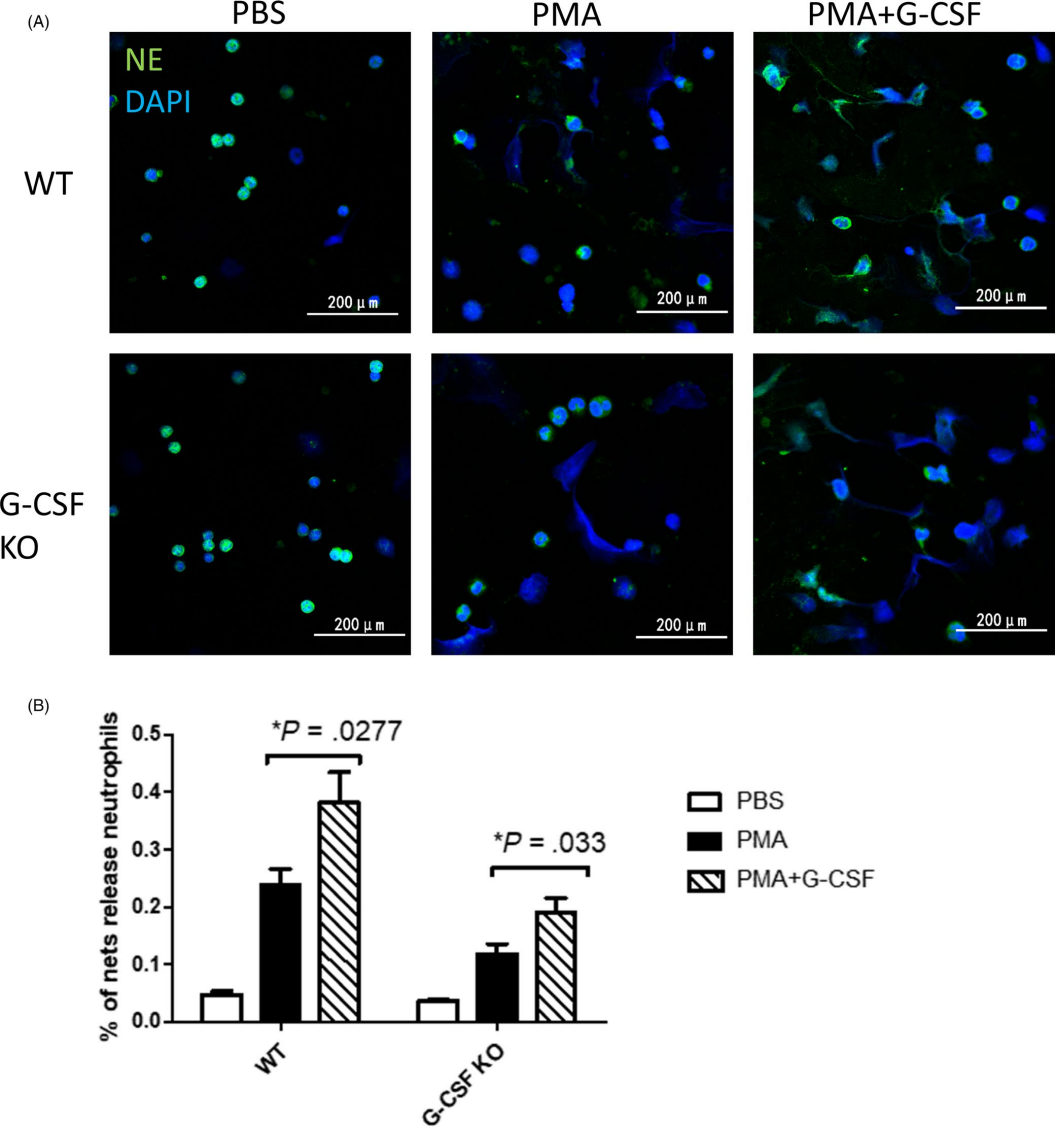
Granulocyte colony‐stimulating factor (G‐CSF) affects the ability of bone marrow (BM) neutrophils to form neutrophil extracellular traps (NETs) in vitro. A, Representative confocal images of immunofluorescent staining of NETs in BM neutrophils isolated from WT and G‐CSF−/− mice with the induction of PBS, phorbol 12‐myristate 13‐acetate (PMA), PMA + G‐CSF. B, Percentage of neutrophils forming NETs per field in WT neutrophils as compared to G‐CSF−/− neutrophils with the induction of PBS, PMA, PMA + G‐CSF, as determined by two‐tailed, unequal variance Student's *t* test (mean ± SEM). Results shown are representative of at least eight fields in different regions for each condition and represent three independent experiments

Then, we observed the formation of NETs stimulated by PMA with or without G‐CSF induction in neutrophils from WT and G‐CSF−/− mice. We found that a lower proportion of G‐CSF−/− neutrophils released NETs compare to WT neutrophils after induction of PMA (Figure 7A,B). With G‐CSF induction, BM neutrophils from both WT and G‐CSF−/− mice showed enhanced NETosis (Figure 7A,B). These results show that neutrophils derived from BM lacking G‐CSF have diminished ability of NETs formation in vitro, and G‐CSF induction can enhance its capacity of NETs formation.Therefore, G‐CSF has a role in NETosis of BM neutrophils.

## 4 DISCUSSION

Bone marrow edema is an early imaging manifestation of RA.^19^ BME score is closely related to the erosion of cartilage and bone in RA and is a sensitive indicator of the risk and progression of bone erosion.^5^ From the perspective of pathology, BME in inflammatory joints of RA indicates abnormal aggregation of T or B lymphocytes in this area, replacing normal adipose tissue, but its exact role and mechanism are still unclear.^9^ Neutrophils are differentiated and mature in the BM. In the state of inflammation, neutrophils can infiltrate in large numbers in the joint cavity of RA after chemotaxis and participate in joint inflammation.^20^ However, whether neutrophils participate in BML of RA has not been reported.

In this study, we used a mouse model of CIA to confirm the presence of significant neutrophil infiltration in subchondral BML of inflammatory joints. Further CIA induction experiments on G‐CSF−/− mice showed that G‐CSF−/− mice had no BML formation and neutrophil infiltration, and arthritis induction was completely blocked. Therefore, G‐CSF‐dependent neutrophil overproduction in CIA mice may lead to numerous neutrophil infiltration in BML. Based on the discovery that the citrullinated histone produced by neutrophils during NETosis is the authentic antigen of ACPA^18^, ^21^ and the existed correlation between ACPA and BME,^6^, ^22^ we speculated that the BM neutrophils were involved in inflammatory arthritis via NETosis. Further immunofluorescence staining results confirmed our speculation that not only the infiltration of NETs in the joints, but also the more significant neutrophils with citrullinated histone in the BM, but no definite NETs formation was observed. Flow cytometry analysis of bone marrow neutrophil maturity showed that there was a neutrophil maturation defect in the bone marrow of G‐CSF−/−mice. The results of inducing BM neutrophils to form NETs in vitro further indicate that NETosis of BM neutrophils is regulated by G‐CSF. Therefore, these results suggest that bone marrow neutrophils are overproduced and primed for NETosis in a G‐CSF dependent manner, which is a critical feature of subchondral BML in the CIA model.Although most researchers focus on the lesions of the synovium, cartilage, and bone cortex, in fact, the subchondral BM also has an important role in RA. McQueen et al found that compared with non‐BME areas, BME areas have a significant inflammatory response, and called this osteitis.^23^ In the CIA model, BM mesenchymal cells can pass through the foramen of the bone cortex into the synovium and produce inflammatory mediators before the onset of arthritis symptoms.^24^ These findings are consistent with the clinical phenomenon that BME can occur before synovial lesions. McQueen et al proposed the pathogenesis hypothesis of RA BML, suggesting that pathological cells can migrate from subchondral bone to synovium to play a role.^10^ Our study reveals that the overproduced BM neutrophils in CIA mice are primed for NETosis, blocking the excessive production of BM neutrophils can inhibit the occurrence of inflammatory arthritis through suppressing the process of BML and NETosis. Therefore, we suppose that these pathological cells within BML play a key role in the development of arthritis, providing new clues for elucidating the role of BME in the pathogenesis of RA.

Neutrophils are terminally differentiated cells and unable to further proliferate. In the absence of stimuli, neutrophils have a very short life span in the circulation (8‐20 hours) before undergoing apoptosis.^25^ In RA patients, it can quickly migrate and infiltrate into the inflamed joint.^11^ The number of neutrophils in the joint cavity is extremely high.^26^ The neutrophils in the synovial fluid of RA patients reach 25 000/mm^3^, accounting for about 90% of the total number. Previous studies have confirmed that neutrophils in the inflamed joints have delayed apoptosis and prolonged life span compared to peripheral blood neutrophils.^27^ Meanwhile, numerous neutrophils promote arthritis through enhanced death such as NETosis.^28^ The excessive production of neutrophils in the BM under the stimulus of inflammation guarantees the continued large demand for neutrophils in the inflammation site, which is particularly critical to maintaining the survival and elimination balance of neutrophils immersed in the inflammatory joints.

Neutrophils infiltrated into inflammatory joints are further involved in arthritis by phagocytosis, degranulation, and production of ROS.^11^ In recent years, the discovery of a new cell death method of NETs has further clarified the role of neutrophils in the pathogenesis of RA. NETs are extracellular structures composed of DNA and granular proteins released from activated neutrophils.^29^ NETs can strongly stimulate synovial fibroblasts or macrophages to express pro‐inflammatory cytokines, while multiple antibodies and inflammatory cytokines can induce NETosis and thus form a vicious cycle.^30^ Current studies have focused on the formation and role of NETs in organs or peripheral blood. To our knowledge, we reveal for the first time that BM neutrophils are primed for NETosis in the CIA model and these cells are prone to form NETs when stimulated by serum from CIA mice. These phenomena mimic the role of the BM neutrophils in vivo in the pathogenesis of CIA. Some literature has reported that serum rich in inflammatory cytokines and autoantibodies from individuals with different diseases can induce NETs formation in vitro,^30^, ^31^, ^32^, ^33^ while normal serum may inhibit NETs formation.^34^ Different results of NETs induction by different serum may also indicate the role of environmental components in neutrophil activation. Besides, the NETosis primed BM neutrophils in our study may provide clues for why there is a correlation between BME and ACPA.

Although the emergence of biological agents has greatly improved the current status of RA treatment, considerable RA patients still do not achieve a satisfactory response, and new therapeutic targets and strategies need to be found. Our study illustrates the role of BM neutrophils in the pathogenesis of RA, and several animal experiments have shown that elimination and inhibition of neutrophils can almost completely inhibit the occurrence of inflammatory arthritis. Properly inhibiting the excessive production or NETosis of these pathological BM neutrophils, may help control the inflammatory process of RA. G‐CSF is a hematopoietic growth factor and a pro‐inflammatory factor. In the case of inflammation, G‐CSF can increase rapidly, and effectively promote the differentiation and release of BM neutrophils. G‐CSF plays an important role in the chemotaxis and infiltration of neutrophils into sites of inflammation, and these effects have been demonstrated in the previously reported arthritis model of G‐CSF knockout mice^35^, ^36^ and the recently reported periodontitis mouse model.^37^ In contrast, our study highlights the role of G‐CSF on BM neutrophil production and NETosis induction in the CIA model. Our results suggest that inhibition of overproduction and NETosis induction of BM neutrophil by targeting G‐CSF may affect the onset of arthritis. Previous studies have suggested that granulocyte maturation determines the ability to release NETs,^38^ which may explain the regulatory effect of G‐CSF on NETs. Nevertheless, the exact mechanism of BM neutrophils primed for NETosis in the CIA model remains unclear. NETosis caused by different stimuli may have different molecular mechanisms and upstream pathways, including NOX‐dependent and NOX‐independent mechanisms.^39^, ^40^ In addition to citrullinated histones, ROS is also an important marker of NETosis. ROS、Ca^2+^ and PAD4 are key participants in NETosis and they are also the focus of in‐depth study for the exact mechanism of BM neutrophils primed for NETosis in CIA.

**FIGURE 8:**
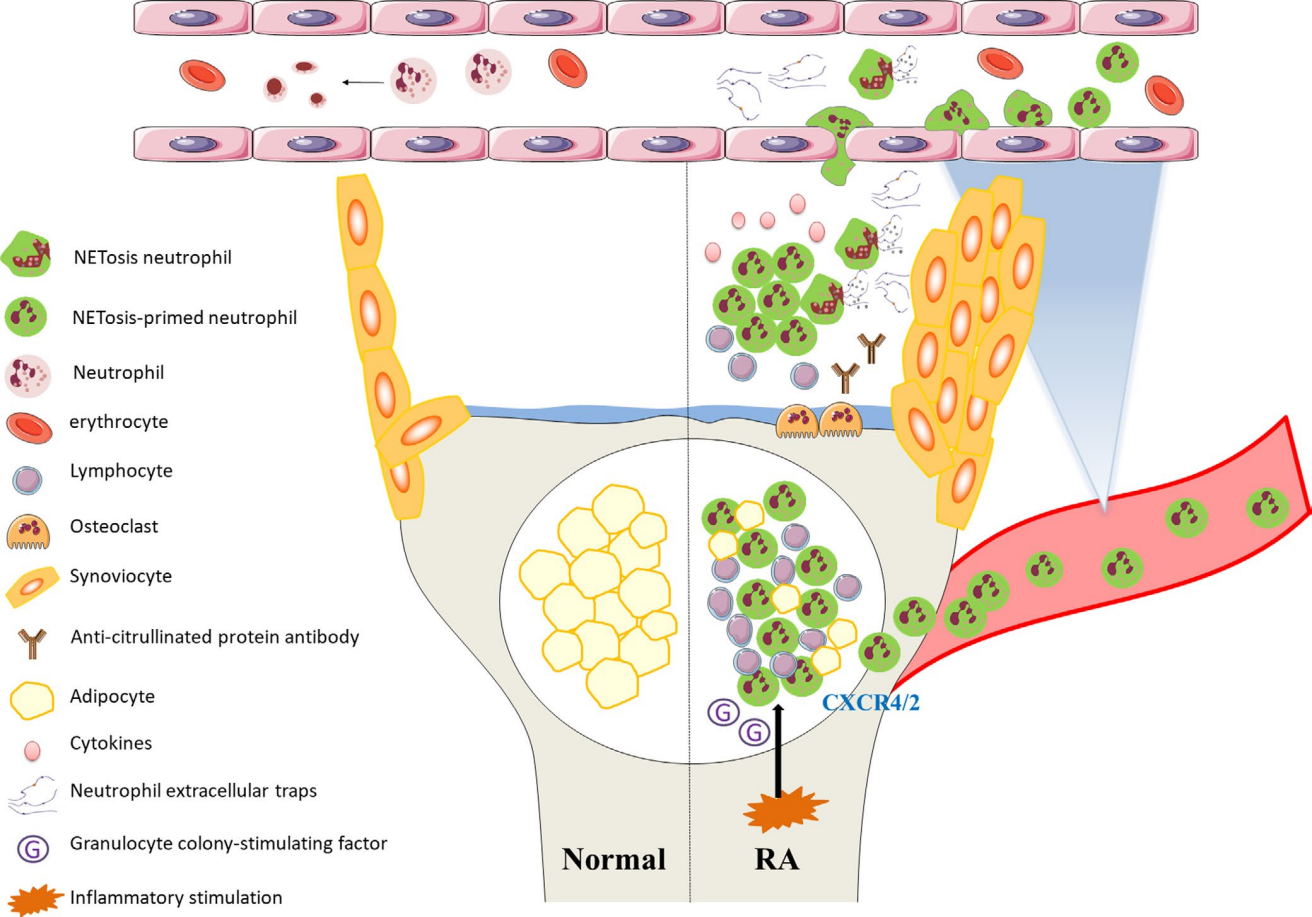
Under inflammation, bone marrow (BM) neutrophils are produced in large quantities under the action of granulocyte colony‐stimulating factor (G‐CSF), meanwhile primed for NETosis. Once released into the periphery and subjected to chemotaxis of inflammation, these pathological cells can infiltrate into inflammatory joints and form neutrophil extracellular traps (NETs), promoting joint inflammation and autoantibody production, eventually leading to bone erosion

In conclusion, overproduced BM neutrophils are primed for NETosis in BM of CIA mice in a G‐CSF dependent manner, and these pathogenic cells may have an important role in initiating and promoting the development of inflammatory arthritis, especially in bone erosion (Figure 8). In the future, more in‐depth and detailed researches are needed to clarify the role and exact mechanism of these cells within the development of RA and further clarify the regulatory mechanism of these pathological BM neutrophils. The crosstalk between BM neutrophils and inflammatory arthritis is an interesting topic worthy of further study. Targeting this pathological process may be a potential strategy for the treatment of RA.

## CONFLICT OF INTEREST

The authors declare that they have no conflict of interest.

## AUTHOR CONTRIBUTIONS

Danyi Xu, Jinming Shen, Chaohui Yu and Jin Lin conceived and designed the study. Danyi Xu conducted the majority of the experiments. Jie Zhang, Jinghua Wang, Yuwei Zhang, Hong Zhang, Longgui Ning, Peihao Liu, Sha Li, Yiming Lin, and Hang Zeng participated in the experiments and data collection. Danyi Xu and Jinming Shen analyzed the data and wrote the manuscript. Yiming Lin designed and conducted the supplementary experiments for revision, wrote the relevant content and responded to the reviewers' comments. Chaohui Yu and Jin Lin supervised the study and revised the manuscript.

## Data Availability

The data that support the findings of this study are available from the corresponding author upon reasonable request.
